# A Comparative Study on Immune Protection Efficacy: An HSV-1 Trivalent Antigen Subunit Vaccine Formulated with a Cellular Immunity-Inducing Adjuvant Versus an mRNA Vaccine

**DOI:** 10.3390/vaccines13090958

**Published:** 2025-09-10

**Authors:** Han Cao, Jingping Hu, Fengyuan Zeng, Ning Luan, Dandan Gao, Zhentao Lei, Jishuai Cheng, Cunbao Liu

**Affiliations:** 1 Institute of Medical Biology, Chinese Academy of Medical Sciences and Peking Union Medical College, Kunming 650118, China; caohan@imbcams.com.cn (H.C.); hujingping@student.pumc.edu.cn (J.H.); zengfengyuan0120@163.com (F.Z.); luanning@imbcams.com.cn (N.L.); ddgao2008@imbcams.com.cn (D.G.); s2023018021@pumc.edu.cn (Z.L.); 2 Laboratory Animal Department, Kunming Medical University, Kunming 650500, China

**Keywords:** HSV-1, subunit vaccine, mRNA vaccine, CMI

## Abstract

**Background:** Herpes simplex virus (HSV) is a neurotropic virus that can be categorized into two serotypes: HSV-1 and HSV-2. HSV-1 causes symptoms such as herpes labialis, herpetic keratitis, genital ulcers, and encephalitis, and primarily establishes latent infection in the trigeminal ganglion. The complexity of membrane fusion mechanisms and potential infection in nerves allow HSV to easily evade recognition and clearance by host immune cells. Therefore, developing a vaccine that can prevent both primary and reactivated HSV-1 infection is critical. Currently, no preventive or therapeutic HSV-1 vaccines have been approved for marketing. **Methods**: In this study, we utilized the gC, gD, and gE proteins of HSV-1, which are associated with viral fusion and immune escape, to design a trivalent antigen vaccine that is capable of inducing a cellular immune response. Two formulations of the vaccine are available: a subunit vaccine incorporating oligodeoxynucleotides with CpG motifs (CpG ODNs) and QS-21 as adjuvants, as well as an mRNA vaccine. Mice were immunized via intramuscular injection to evaluate and compare the immunological responses and protective efficacy of the two vaccines. **Results**: After the challenge, the viral load in the tissues of both vaccine groups was significantly lower than that in the positive control group, indicating that both vaccines were able to control viral proliferation in the tissues. **Conclusions**: The findings indicated that both mRNA and subunit vaccines were capable of eliciting comparable humoral and cellular immune responses.

## 1. Introduction

The World Health Organization (WHO) estimates that more than 4 billion people are infected with the herpes simplex virus (HSV). Among these, approximately 3.7 billion are infected with type 1 (HSV-1), while approximately 400 million are infected with type 2 (HSV-2) [1]. HSV-1 is particularly prevalent in the Americas, Europe, and the western Pacific, where it is more common than HSV-2. HSV-1 infection can lead to inflammation of the lips, known as herpes labialis. When the infection is severe, it can induce conjunctivitis, which is a primary cause of infectious blindness in developed countries [2]. Moreover, emerging evidence suggests that recurrent HSV-1 infections may be causally linked to neurodegeneration in patients with Alzheimer’s disease [3]. Most importantly, HSV-1 can invade nerve cells and cause damage to the nervous system, resulting in herpes simplex encephalitis [4]. Moreover, HSV-1 usually survives in the host body as a form of latent infection for a long time. This state allows the virus to evade the host’s immune system [5]. The human trigeminal ganglion is a major latent site for HSV-1 [6], and eliminating the virus once it is established is difficult [7].

HSV-1 belongs to the family Herpesviridae and the alpha-Herpesviridae subfamily [8]. HSV-1 has one of the most complex viral genomes, consisting of a 152-kilobase (Kb) linear double-stranded DNA molecule that encodes at least 83 viral proteins [9]. Among these proteins, the glycoproteins gB, gC, gD, gE, gH, and gL on the outermost lipid bilayer envelope of HSV-1 are crucial for virus entry into host cells and viral immune escape [10,11]. gD is an important receptor-binding protein that recognizes and binds to specific receptors on the host cell surface [12,13,14], such as nectin-1. This interaction triggers the membrane fusion cascade during viral entry [15]. The gD protein is also the most abundant glycoprotein present on the surface of the viral membrane and contains several neutralizing epitopes [16], making it a common target for vaccine development. The gC protein is involved in immune escape through binding to the complement component C3b, which inhibits complement activation [17]. Moreover, gC mediates the binding of the virus to heparan sulfate on the cell surface, which is the initial step in viral adsorption. The glycoproteins gE and gI exist as heterodimers [18] and play important roles in the viral life cycle. The gE protein can bind the Fc domain of IgG [19], which interferes with C1q-binding antigen-antibody complexes, thereby blocking complement activation and antibody-dependent cell-mediated cytotoxicity (ADCC) activities [20,21]. In summary, the glycoproteins gC, gD, and gE are potential antigen targets with a variety of antiviral activities, including neutralizing viruses (gC and gD), blocking cell-to-cell spread (gD and gE), and preventing immune evasion by antibodies and complement (gC and gE). Harvey M. Friedman’s team suggested that targeting the immune escape zones of gC and gE may be a promising direction for HSV vaccine research [22]. Therefore, incorporating gC and gE as immunogens in addition to gD in HSV vaccine formulations may enhance the protective efficacy of the vaccine [23,24,25].

Currently, there are no licensed HSV vaccines for herpes simplex virus; however, several vaccines are currently in clinical trials [1], including Modena’s mRNA-1608 and BioNTech’s BNT162b2, both of which target HSV-2. One of the major problems with the development of HSV vaccines is the complex interactions between the immune response and the virus. mRNA formulated with an ionizable lipid nanoparticle (LNP) delivery system is a novel vaccine strategy that possesses robust immunogenic properties and acts as a self-adjuvant, which can activate both humoral and cellular immune responses [26,27,28]. LNPs are composed of four different lipids with specific functions: ionizing cationic lipids form electrostatic complexes with anionic DNA and RNA to promote intracellular uptake and endosomal escape; cholesterol contributes to complex stability and mobility; phospholipids enhance structural integrity; and pegylated lipids maintain stability, achieve cellular uptake, and protect lipid-DNA and RNA complexes from protein corona capture [29]. Despite the abovementioned priorities, the manipulation of mRNA requires very strict environments, and compared with other vaccines, mRNA vaccines require much more harsh cold chains. Therefore, we designed a subunit vaccine for HSV-1 and compared the immune effects of the mRNA and subunit vaccines in this study.

However, protein subunit vaccines exhibit limited immunogenicity and may not effectively elicit specific cellular immune responses. In our previous research, we demonstrated that the combination of oligodeoxynucleotides containing CpG motifs (CpG ODNs) and QS-21 could enhance the cell-mediated immunity (CMI) of a VZV subunit vaccine [30,31]. To increase CMI, CD8(+) T cells and host cellular factors are crucial for controlling viral latency [32]; thus, we utilized CpG ODNs [33] and QS-21 [34] as adjuvants in the subunit vaccine. In this study, we compared the immunoprotective efficacy of mRNA and subunit vaccines targeting these three antigens.

## 2. Materials and Methods

### 2.1. Vaccine Preparation

Two types of vaccines containing three antigens, namely, mRNA and subunit vaccines, were prepared in this study. The grouping of the vaccines and the dosing of the three antigens are shown in Table 1. The subunit vaccine consisted of gC1, gD1, and gE1 proteins and adjuvants that were mixed before immunization with PBS. The three protein pathogens were prepared by AtaGenix Laboratory Co., Ltd., Wuhan, China. In brief, the target fragments of the proteins were cloned and inserted into the pATX2 vector, and a 6×His tag was added to the C-terminus of the target fragment. Chinese hamster ovary (CHO) cells were used to express the proteins, and a nickel column was used to purify the proteins. The CpG ODN 1018S used in the vaccine was synthesized by Sangon Biotech (Shanghai, China, CHN). QS-21 was supplied by Alpha Diagnostic Intl. Inc. (San Antonio, TX, USA).

Raw materials for the preparation of the mRNA vaccine were provided by Nanjing Vazyme Biotech Co., Ltd. (Nanjing, China). The synthetic DNA sequence was transcribed into mRNA in vitro and purified using magnetic beads, after which the mRNA concentration was detected using the Quant-iT™ RiboGreen^®^ RNA Reagent and Kit (Thermo Fisher, Waltham, CA, USA) [35]. Lipids (from AVT Pharmaceutical Technology Co., Ltd., Shanghai, China) were dissolved in ethanol at the following molar ratio: ionizable lipid (MC3): 1,2-distearoyl-sn-glycero-3-phosphocholine(DSPC):cholesterol:1,2-dimyristoyl-rac-glycero-3-methoxypolyethylene glycol-2000 (DMG-PEG2000) = 46.3:9.4:42.7:6. A microfluidic device (Precision Nanosystems, Vancouver, BC, Canada) was used to encapsulate the mRNA in the lipid phase at a ratio of 3:1 (water:lipid). The liposomes were concentrated using an ultrafiltration tube (Merck Millipore, Burlington, MA, USA) containing PBS solution (treated with DEPC). A Malvern ZEN3600 (Malvern Instruments Ltd., Worcestershire, UK) was used to measure the size and polydispersity index (PDI) of the mRNA vaccine. Encapsulation of mRNA was detected using 1% denaturing agarose gel electrophoresis, and the nucleic acid load of the mRNA was quantified using the Quant-iTTM RiboGreen^®^ RNA Reagent and Kit (Thermo Fisher Scientific, Pleasanton, CA, USA). The encapsulation efficiency was determined by comparing the quantified nucleic acid load to the initial amount of nucleic acid added to the citric acid buffer.

### 2.2. Mouse Study Design

HSV-1-specific pathogen-free female BALB/c mice, aged 5–6 weeks, were raised in a specific pathogen-free (SPF) environment at the Small Animal Experiment Department of the Institute of Medical Biology, Chinese Academy of Medical Science (IMBCAMS).

For the immunization experiment, the mice were divided into 3 groups, with 18 mice in group 1 and 12 mice in groups 2 and 3. All the mice in the vaccine group were immunized with two doses with an interval of 4 weeks between doses. The mice in groups 2 and 3 were immunized intramuscularly with a total volume of 50 μL containing 15 μg of antigen in PBS, and the mice in group 1 were intramuscularly immunized with 50 μL of PBS. Two weeks after the second immunization, 6 mice from each group were selected for blood collection and spleen harvesting. Whole blood was collected from the heart, and the spleens were removed and treated as described previously [31] to obtain a single-cell suspension of spleen cells.

To assess the protective effect of the vaccines against the HSV-1 virus, after 28 days of immunization (the mice are listed in Table 1), the remaining mice (not used for blood and spleen collection) in each group were challenged with the HSV-1 Mckrae wild-type strain via nasal drops at a dose of 2 × 10^4^ PFU [36]. In group 1, 6 mice were challenged with the wild-type strain as a positive control, and 6 mice were not challenged as a negative control. The behavior of the mice was observed for 10 consecutive days, possible clinical symptoms were recorded, and the survival rate of the mice was calculated. Finally, mouse tissue samples were taken and stored at −80 °C.

### 2.3. Specific IgG Titer Detection

An indirect enzyme-linked immunosorbent assay (ELISA) was used to detect the specific IgG titers. The collected blood was placed at 4 °C overnight and then centrifuged at 3000 rpm for 20 min to separate the serum. A volume of 100 μL of the HSV-1 proteins C1, D1, and E1 (each at a concentration of 2 μg/mL) was precoated onto 96-well plates (Corning, Corning, NY, USA) at 4 °C overnight. After removal from the 4 °C environment, the plates were washed with PBST to remove unbound proteins. To block the unbound sites, 5% (*w*/*v*) skim milk was added to the plates, which were subsequently incubated at 37 °C for 1 h. Two-fold serial dilutions of mouse serum (diluted from 1000 to 2,048,000) were subsequently added to the plates and incubated at 37 °C for 1 h. After the samples were washed with PBST, a goat anti-mouse IgG-horseradish peroxidase (HRP) conjugate (1:10,000; Bio-Rad, Hercules, CA, USA) was added as a secondary antibody and incubated at 37 °C for 1 h. Afterward, the plates were washed with PBST, and the substrate 3,3′,5,5′-tetramethylbenzidine (TMB; BD, San Diego, CA, USA) was added at room temperature for 5 min. After incubation, 100 μL of 2 mol/L sulfuric acid was added to terminate the reaction. The absorbance at 450 nm was measured using a spectrophotometer (BioTek Instruments, Inc., Winooski, VT, USA). IgG titers were defined as the maximum dilutions of serum with a cutoff signal above OD450 = 0.15, and the antibody titer of samples with an absorbance less than 0.15 at 450 nm was recorded as 100.

### 2.4. Enzyme-Linked Immunosorbent Assay of Splenocyte-Secreted Cytokines

ELISAs with the double-antibody sandwich method were used to measure the levels of the cytokines IL-2 and IFN-γ. Spleen cells were obtained by grinding the mouse spleen, and Roswell Park Memorial Institute (RPMI) 1640 medium was added to adjust the cell concentration to 1 × 10^7^ cells/mL. Afterward, 100 μL of the spleen cells (1 × 10^6^ cells per well) was added to each well, and 50 μL of the gC1, gD1, and gE1 protein mixture (15 μg/mL) was added and cultivated at 37 °C with 5% CO_2_ for approximately 24 h. Positive and negative control groups were also established, with the positive control group stimulated with PMA+. After incubation, the supernatants were collected, and the cytokine levels were detected using the ELISA standard method.

Unconjugated anti-IL-2 (3 μg/mL) and anti-IFN-γ (4 μg/mL) antibodies (Invitrogen, Waltham, MA, USA) were dissolved in PBS and coated onto 96-well plates at 4 °C overnight. After the plates were washed three times with PBST, they were blocked with 1% (*w/v*) BSA at 37 °C for 1 h, after which 50 μL of the cell supernatant and standard protein were added to each well and incubated for 3 h at room temperature. After the cells were washed, biotin-conjugated antibodies against IL-2 and IFN-γ (2 μg/mL; Invitrogen) dissolved in 1% BSA were added to detect the cytokines. The plates were incubated at 37 °C for 1 h, followed by the addition of HRP-conjugated streptavidin (1 μg/mL; Biolegend, San Diego, CA, USA) and incubation for 30 min at 37 °C. Finally, TMB substrate (BD, San Diego, CA, USA) was added for color rendering, and 2 mol/L of sulfuric acid was added to terminate the reaction on the basis of the color change in the wells.

### 2.5. Enzyme-Linked Immunospot (ELISPOT) Assay of Splenocytes

ELISPOT plates (Merck, Rahway, NJ, USA) were used to measure the levels of the cytokines IL-2 and IFN-γ. The plates were activated with 75% ethanol and subsequently washed with PBS to remove residual ethanol. The plates were coated with specific IL-2 and IFN-γ antibodies (Invitrogen, Waltham, MA, USA) at a concentration of 2 μg/mL (100 μL/well) and incubated at 4 °C overnight. Following this, the cells were washed with complete 1640 medium, and fresh 1640 medium containing 10% FBS was added to block the uncoated sites for 2 h. The wells were then filled with 30 μL of cells (containing 3 × 10^5^ spleen cells), supplemented with 70 μL of 1640 medium and 50 μL of cell stimulus (a mixture of gE1, gD1, and gC1 proteins, 30 µg/mL; 1:1:1 ratio). A negative control group and a positive control group were established, with PMA+ included in the latter. The 96-well plates were cultured overnight at 37 °C and 5% CO_2_ without movement. After centrifugation at 800× *g* for 5 min, cold deionized water and PBS were used to clean the wells. Subsequently, secondary antibodies against IL-2 and IFN-γ (1 μg/mL; Invitrogen, Waltham, MA, USA) were added to each well, and the plates were incubated at room temperature for 3 h. Diluted HRP-conjugated streptavidin (1:1500; Biolegend, San Diego, CA, USA) was then added and incubated for 1 h at room temperature. Finally, an ELISPOT assay kit (Dakewe Biotech Co., Ltd, Guangdong, China) was used to visualize the spots according to the manufacturer’s protocol, and the reaction was stopped using flowing water. The spots were counted according to the manufacturer’s protocol (Autoimmun Diagnostika GmbH, Strassberg, Germany).

### 2.6. Flow Cytometry

The treated spleen cells (1 × 10^6^ cells per well) and stimulus (a mixture of gE1, gD1, and gC1 proteins, 21 µg/mL; 1:1:1 ratio) were added to 24-well plates (Corning) and incubated at 37 °C for 2 h. Brefeldin A was then added and incubated overnight to block cytokine release at a final concentration of 5 μg/mL. The cell suspension was transferred to a 1.5 mL centrifuge tube (NEST Biotech Co., Ltd, Jiangsu, China), and the 24-well plates were subsequently washed with 500 μL of PBS. Zombie NIR™ dye with 100 μL of DMSO was subsequently diluted with PBS at a dilution ratio of 1:2000, after which 100 μL of the diluted dye was added to each centrifuge tube. The tubes were incubated at room temperature in the dark. After the samples were washed twice with staining buffer, the nonspecific binding of Fc receptors was blocked using 5 μg/mL CD16/CD32 antibodies. Next, a mixture of PerCP/Cyanine 5.5-tagged anti-mouse CD4 and FITC-tagged anti-mouse CD8 antibodies was added and incubated at 4 °C for another 30 min. After being washed with staining buffer, the cells were fixed with 4% formaldehyde and incubated at room temperature in the dark for 20 min to stabilize the cell membrane. After the cells were washed twice with permeabilization wash buffer, intracellular staining was performed using PE-conjugated anti-mouse IFN-γ and APC-conjugated anti-mouse IL-2 antibodies dissolved in permeabilization wash buffer. The cells were incubated at room temperature in the dark for 40–60 min. Following staining, a CytoFLEX flow cytometer (Beckman, Indianapolis, IN, USA) was used to analyze the CD4+ T and CD8+ T cells in the samples. All the reagents used in the experiments were obtained from BioLegend (San Diego, CA, USA). A Flowjo™10.8.1 (BD, San Diego, CA, USA) was utilized for the analysis of flow cytometry data. First, the lymphocytes were gated based on their forward scatter (FSC-A) and side scatter (SSC-A) characteristics. Then, dead cells were excluded using Zombie NIR™ dye. Subsequently, CD4+ and CD8+ T cell populations were identified from the live lymphocytes. Finally, the percentages of IL-2- and IFN-γ-positive cells within the CD4+ and CD8+ T cell subsets were analyzed.

### 2.7. Serum Neutralizing Antibody Titer Detection

A neutralization assay was performed in accordance with standard protocols as described previously [37]. Briefly, the mouse serum was inactivated at 56 °C for 30 min, after which the serum was serially diluted in 96-well plates at a ratio of 1:4:8:16:32:64:128 (3 replicates). A negative control group lacking the virus and serum was established, and a positive control group with only the virus was established. Serum dilutions were incubated with the HSV-1 virus for 2 h at 37 °C. After incubation, the virus–serum mixture was added to Vero cells and incubated at 37 °C for 4–7 days. Subsequently, the cytopathic effect (CPE) was observed daily under a microscope to determine the neutralizing antibody titer of each serum sample. The geometric mean titers (GMTs) were expressed as the geometric mean titer (GMT) ± standard deviation (SD).

### 2.8. Copy Number of the HSV-1 Virus in Mouse Tissues Detected by Real-Time Quantitative PCR

Mouse tissues (the heart, liver, spleen, lungs, kidney, brain, spinal cord, and trigeminal nerve) were ground up. Nucleic acid was extracted from the tissue samples according to the instructions of the TaKaRa MiniBEST Viral RNA/DNA Extraction Kit Ver.5.0 (TaKaRa, Tokyo, Japan). Afterward, the HSV-1 viral load in the tissue samples was detected by the Taqman probe method with a real-time fluorescence quantitative PCR kit (TaKaRa). We designed specific primers and probes targeting highly conserved regions of the gG gene (Table 2).

### 2.9. Histopathological Hematoxylin—Eosin (HE) Staining

The brains and spinal cords of the mice were fixed in tissue fixation fluid (Servicebio, Wuhan, China) and embedded in paraffin in tissue blocks. Approximately two slides per organ were stained with hematoxylin and eosin (H&E) to assess the morphology. The thickness of the paraffin section was 4 μm.

### 2.10. Statistical Analysis

GraphPad Prism 9.5.1 (GraphPad Software, La Jolla, CA, USA) was used for the data analyses. Significant differences among experimental groups were analyzed using ordinary one-way analysis of variance (ANOVA), followed by Tukey’s multiple comparisons test to compare the mean of each group with the mean of every other group. The scatter plots were plotted as mean with SD.

Statistical significance was determined as follows: *p* > 0.05 (nonsignificant, ns), *p* ≤ 0.05 (significant, *), *p* ≤ 0.01 (highly significant, **), *p* ≤ 0.001 (extremely significant, ***), and *p* ≤ 0.0001 (extremely significant, ****).

## 3. Results

### 3.1. LNPs Efficiently Encapsulate mRNA with a Uniform Particle Size

**Figure 1:**
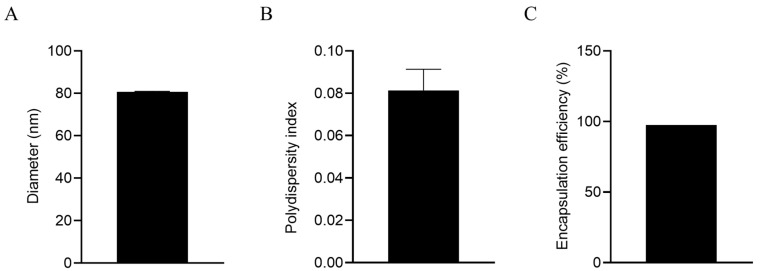
Characterization of LNP mRNA vaccines. (**A**) Diameter tested by a size analyzer; (**B**) polydispersity index of LNPs; (**C**) mRNA encapsulation efficiency.

The diameter of the LNPs encapsulating gC1, gE1, and gD1 mRNA was 80.67 nm, with a PDI of 0.081, indicating good uniformity of the nanoparticles. After being lysed with 0.1 mol/L NaOH containing 0.1% SDS (*m*/*v*) overnight at 4 °C, the mean mRNA encapsulation efficiency was 97.4%, indicating that the mRNA in the LNP vaccine exhibited minimal degradation, indicating good integrity (Figure 1).

### 3.2. IgG-Binding Antibodies and Neutralizing Antibodies Induced by Different Vaccines 

**Figure 2:**
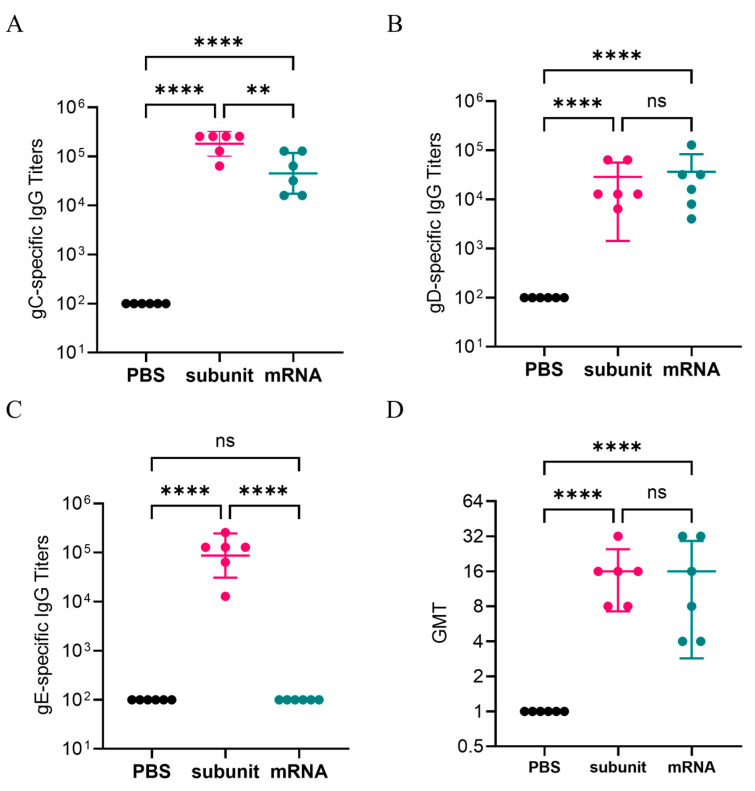
gC1, gD1, and gE1-specific IgG titers detected by ELISA. Neutralizing antibody titers. (**A**) gC1-specific IgG responses. (**B**) gD1-specific IgG responses. (**C**) gE1-specific IgG responses. (**D**) Neutralizing antibody titers of mouse serum samples. The Y-axis represents the dilution of serum. The differences between groups are indicated as follows: ns, nonsignificant difference with *p* > 0.05; **, significant difference with *p* ≤ 0.01; ****, significant difference with *p* ≤ 0.0001.

After two doses of immunizations, gC1-, gD1-, and gE1-specific IgG antibodies were detected by indirect ELISA. The results (Figure 2B) indicated that the titers of IgG-specific antibodies against the gD1 proteins induced by the subunit vaccine were similar to those in the mRNA vaccine group. However, compared with the mRNA vaccine, the subunit vaccine induced significantly higher titers of gC1- and gE1-specific antibodies (Figure 2A,C). Notably, the subunit vaccine induced higher gE1-specific antibody levels, with an average titer of 1,194,66, whereas the mRNA vaccine appeared to elicit minimal or no detectable gE1-specific antibody response.

In addition, we measured the neutralizing antibodies of the serum samples from three groups: the negative control, mRNA vaccine, and subunit vaccine groups. The results are presented in Figure 2D. The GMTs of the neutralizing antibodies for the mRNA vaccine group and the subunit vaccine group were similar, with values of 11.31 and 14.25, respectively. The results indicated that both the mRNA vaccine and the subunit vaccine may induce pathogen-specific IgG-binding antibodies and positive neutralizing antibodies.

### 3.3. Vaccines Elicited a Certain Level of Cellular Immunity

CMI is a crucial immune factor in HSV-1 infection. The CMI is a crucial indicator for evaluating the protective efficacy of three vaccines. To assess the extent of vaccine-induced cellular immune responses, we used three methods, namely, ELISA, ELISPOT, and flow cytometry, to measure the levels of IL-2 and IFN-γ, which are key cytokines involved in cellular immunity.

#### 3.3.1. Extracellular Cytokine Secretion Was Detected by ELISA and ELISPOT

**Figure 3:**
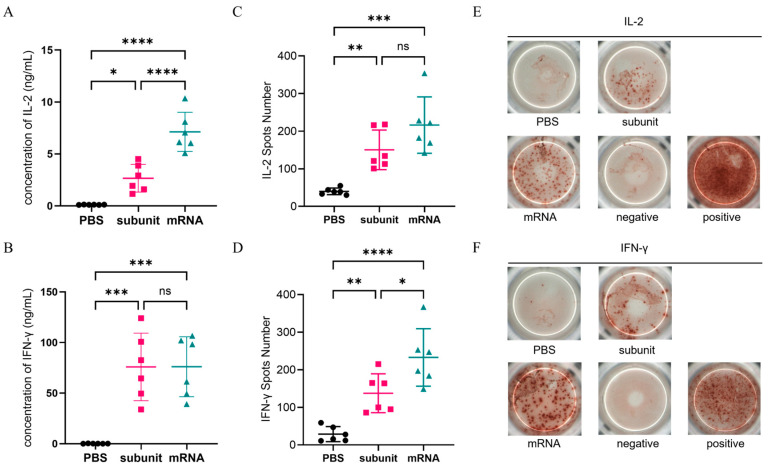
Cellular immune response levels in mice were measured by ELISA and ELISPOT. (**A**,**B**) ELISA detection of the concentrations of IL-2 (**A**) and IFN-γ (**B**) in the supernatants of mouse spleen cells. (**C,D**) ELISPOT was used to detect the number of spots of IL-2 (**C**) and IFN-γ (**D**). (**E,F**) Representative photos of the ELISPOT reaction of IL-2 (**E**) and IFN-γ (**F**). Points represent individual mice. ns: nonsignificant difference with *p* > 0.05; * significant difference with *p* ≤ 0.05; ** significant difference with *p* ≤ 0.01; *** significant difference with *p* ≤ 0.001; **** significant difference with *p* ≤ 0.0001.

ELISA and ELISPOT, which are both specific and sensitive methods for detecting cytokines, were used to detect the secretion of extracellular IL-2 and IFN-γ. The ELISA results (Figure 3A,B) revealed that compared with the subunit vaccine formulated with CpG and QS-21, the mRNA vaccine elicited higher levels of IL-2 (*p* ≤ 0.0001), but there was no significant difference in the IFN-γ levels. Overall, both vaccines demonstrated comparable efficacy in inducing a cellular immune response according to the ELISA results. Additionally, ELISPOT, which detects secreted extracellular cytokines through spot display (Figure 3E,F), further verified the ELISA results (Figure 3C,D). These findings suggest that the subunit vaccine with CpG and QS-21 is capable of eliciting a robust cellular immune response comparable to that elicited by the mRNA vaccine.

#### 3.3.2. The Number of CD4+ T Cells and CD8+ T Cells Capable of Producing IL-2 and IFN-γ Induced by the Vaccines

**Figure 4:**
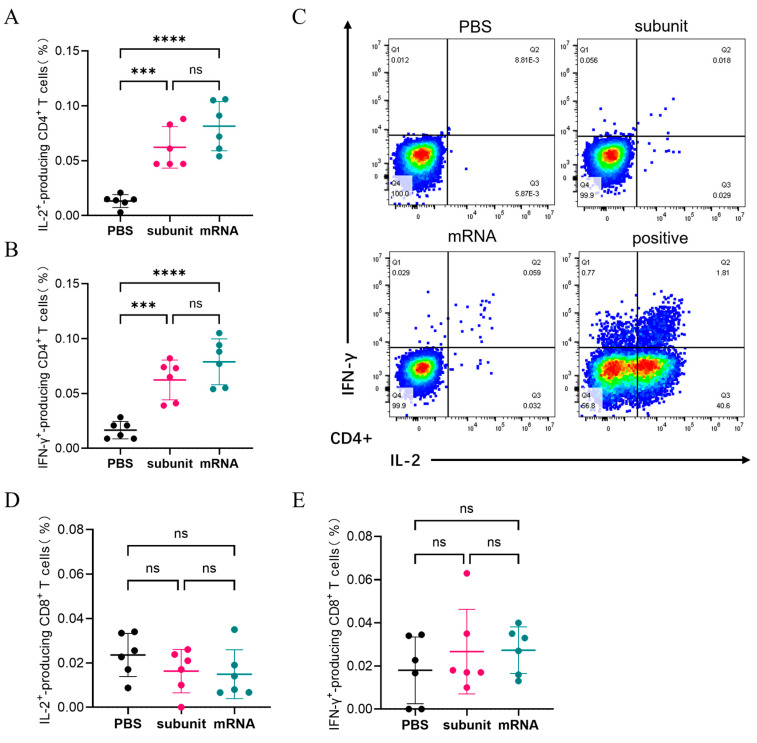
Cellular immune response levels in mice were measured by flow cytometry. (**A**,**B**) The proportion of CD4+ T cells that produce IL-2 (**A**) or IFN-γ (**B**) among mouse spleen cells. (**C**) Representative images of the flow cytometry results. (**D**,**E**) The proportion of CD8+ T cells that produce IL-2 (**D**) or IFN-γ (**E**) among mouse spleen cells. The differences between groups are indicated as follows: ns, nonsignificant differences with *p* > 0.05; ***, significant differences with *p* ≤ 0.001; ****, significant differences with *p* ≤ 0.0001.

Flow cytometry was utilized to detect the intracellular cytokines using several specialized dyes. As shown in Figure 4A,B, the ability of the subunit and mRNA vaccines to induce the production of IL-2 and IFN-γ by CD4+ T cells did not significantly differ. The percentage of CD4+ T cells capable of producing both IL-2 and IFN-γ in response to the subunit vaccine was 0.062%. The proportions of CD4+ T cells that produced IL-2 and IFN-γ in response to the mRNA vaccine were 0.0815% and 0.0788%, respectively. Conversely, both the subunit and mRNA vaccines were less effective at activating CD8+ T cells to produce IL-2 and IFN-γ (Figure 4D,E). The results indicated that the immune effects of the subunit and mRNA vaccines in CMI were similar.

### 3.4. Clinical Symptoms After HSV-1 Infection in Mice

**Figure 5:**
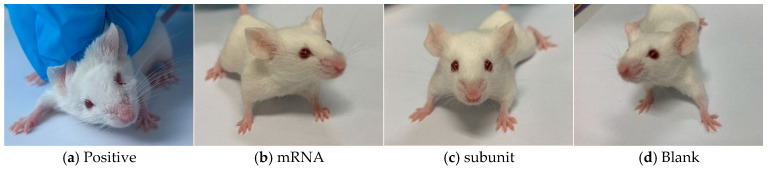
Four pictures of mice in different states.

Following the infection of mice in different groups with the HSV-1 Mckrae wild-type strain, two mice in the positive control group died on day 6, whereas all the mice in the immunized groups survived. Furthermore, compared with the mice in the mRNA group, subunit group, and blank group, the four surviving mice in the positive control group presented obvious symptoms, such as a back bow, hair drooping, keratitis, and decreased mobility (Figure 5). In contrast, the mice in the mRNA group and subunit group did not display any symptoms, and the status of the mice in both groups was as normal as that in the blank group. The above results indicate that immunization with the mRNA and subunit vaccines effectively protected against HSV-1 infection (Table 3).

### 3.5. Viral Load in Different Tissues of Mice After HSV-1 Challenge

**Figure 6:**
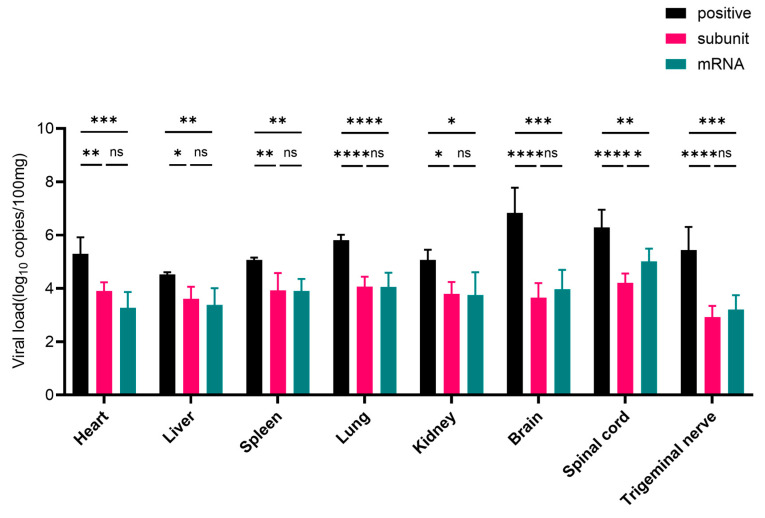
RT-PCR results of viral load in different groups of mouse tissue. The differences between groups are indicated as follows: ns, nonsignificant differences with *p* > 0.05; *, significant differences with *p* ≤ 0.05; **, significant differences with *p* ≤ 0.01; ***, significant differences with *p* ≤ 0.001; ****, significant differences with *p* ≤ 0.0001.

After challenge with the HSV-1 McKrae wild-type strain, heart, liver, spleen, lung, kidney, brain, spinal cord, and trigeminal nerve tissues were collected. Compared with those in the positive control group, the viral loads in the tissues of mice inoculated with the subunit and mRNA vaccines were lower (Figure 6). In the heart, liver, spleen, lung, and kidney tissues, the viral load was approximately 5 times greater than that in the subunit vaccine group, and these differences were statistically significant (*p* < 0.05). Notably, in neural tissues, including the brain, spinal cord, and trigeminal nerve, the viral load in the positive control group was approximately 4400, 190, and 500 times greater, respectively, than that in the subunit vaccine group.

The viral load in the heart, liver, spleen, lung, and kidney tissues of the mRNA group significantly differed from that in the control group (*p* < 0.05), and the viral load in the positive control group was approximately 8 times greater than that in the mRNA vaccine group. More importantly, compared with those in the mRNA vaccine group, the viral loads in the brain, spinal cord, and trigeminal nerve tissues in the positive control group were approximately 1900, 26, and 220 times greater, respectively.

These results further indicate that HSV-1 mRNA and subunit vaccines can inhibit the proliferation of wild-type virus in various tissues and organs of mice, especially in nervous system tissues, suggesting that these two types of vaccines have excellent protective effects. Overall, the subunit and mRNA vaccines exhibited comparable levels of protection. However, the viral loads in nervous system tissues were consistently lower in the subunit vaccine group than in the mRNA group. Notably, in the spinal cord, compared with the mRNA vaccine, the subunit vaccine resulted in a significantly lower viral load (*p* < 0.05), indicating superior neuroprotective efficacy.

### 3.6. Local Histopathology During the Acute Phase of HSV-1 Infection in Mice

The clinical symptoms observed in the mice following the challenge indicated that, compared with the other two vaccine groups, the positive control group exhibited more severe signs of viral infection. Numerous studies have shown that HSV-1 is a neurotropic virus that causes mainly nervous inflammation and damage in mice after infection. Consequently, we collected brain and spinal cord tissues from mice on the sixth day of virus infection for pathological analysis to further understand HSV-1 infection in neural tissue.

**Figure 7:**
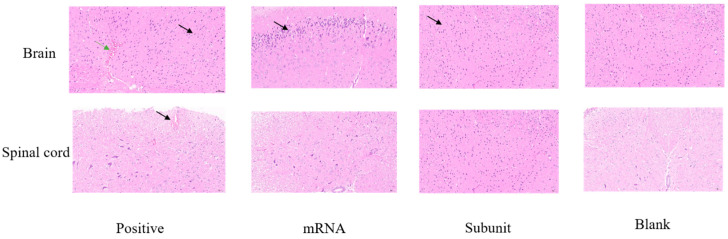
HE staining was used to observe pathological injury to the brain and spinal cord in the different vaccine groups.

Compared with those in the mRNA and subunit groups, neuronal shrinkage was observed in the CA2 region of the cortex and hippocampus in the positive control group (Figure 7). Additionally, the intensity of cell staining increased, the nucleocytoplasmic boundary appeared unclear, and the number of cortical neurons increased (black arrow). Slight localized bleeding was observed in the brain (green arrow), and localized bleeding was also noted in the spinal cord tissue (black arrow). However, both the mRNA and subunit groups of mice exhibited mild pathological changes in their brain tissue. In the mRNA group, a limited number of neurons were found in the cortex of the brain tissue, accompanied by intensified cellular staining and indistinct cytoplasmic boundaries of the nucleus (black arrow). In the subunit group, an increased number of neurons was observed in various regions of the cortex and hippocampus, accompanied by intensified cellular staining and indistinct cytoplasmic boundaries of the nucleus, as indicated by the black arrow. The spinal cord of the mRNA and subunit groups was similar to that of the blank control group, and no notable pathological alterations were observed. These pathological findings further indicated that mice immunized with the HSV-1 mRNA and subunit vaccines can control neural damage, particularly to the brain and spinal cord, from infection and damage caused by the wild-type virus.

## 4. Discussion

In this study, the gC1, gD1, and gE1 proteins were selected as antigens for the design of a trivalent antigen subunit vaccine and mRNA vaccine. Both vaccine platforms induced robust humoral immunity, as evidenced by specific IgG antibodies against the three antigens and effective neutralizing antibodies. Notably, compared with the mRNA vaccine, the subunit vaccine elicited significantly higher gE1-specific IgG titers, which may be attributed to the native-like protein conformation of gE1 in the subunit formulation—facilitating stronger B-cell recognition and antibody production. The reasons why mRNA vaccines fail to induce an effective gE1-specific IgG response in vivo are diverse and may involve mRNA delivery efficiency, stability, translation efficiency, antigen immunogenicity, and immune response kinetics. We need to spend more time on structural research in subsequent studies. In contrast, the comparable neutralizing antibody titers between the two groups suggest that both vaccines successfully targeted critical epitopes involved in viral entry and immune escape. These results are consistent with the known role of gD as a major neutralizing antigen, as gC1 and gE1 proteins are crucial for viral intercellular transmission and immune escape [38]. Specifically, the gE1 protein functions as an Fc receptor and binds to specific IgG in serum to reduce the effect of humoral immunity. These findings suggest that the combination of these three antigens in the form of subunit vaccines or mRNA vaccines is very promising as a prophylactic vaccine.

Moreover, the ELISA and ELISPOT results revealed that both mRNA and subunit vaccines could cause relatively consistent cellular immune responses. The detection indices of the cellular immune response are IL-2 and IFN-γ. IL-2 can activate T cells and promote the production of other cytokines, whereas IFN-γ is an important antiviral substance. The flow cytometry results revealed that the two vaccines stimulated mainly CD4+T cells to produce high amounts of IL-2 and IFN-γ but did not elicit a strong immune response against CD8+T cells. Importantly, the subunit vaccine, when formulated with CpG ODNs and QS-21, achieved cellular immune responses comparable to those of the mRNA vaccine—validating the utility of these adjuvants in enhancing CMI, as observed in previous VZV studies.

In addition, we also conducted a virus protection experiment, and the results revealed that the viral load in the tissues of vaccinated mice was significantly lower than that in the positive control group. No deaths were observed in the vaccine groups. Histopathological analysis further confirmed that compared with control mice, vaccinated mice exhibited minimal neural damage, underscoring the ability of the vaccines to limit neurotropism—a key feature of HSV-1 pathogenesis. The marked reduction in viral load in the trigeminal ganglion, a primary latent site, suggests that both vaccines may interfere with latent viral establishment or reactivation, although long-term studies are needed to confirm this finding. The protective effect of the vaccines is consistent with the results of neutralizing antibody titers and the cellular immune response.

Following the emergence of COVID-19, mRNA vaccines have proven to be highly efficacious immunization strategies. Therefore, in this study, we compared the immune effects of a trivalent subunit vaccine with those of a cellular-mediated immune-inducing adjuvant and an mRNA vaccine. The adjuvant combination of CpG and QS-21 effectively mitigated the inherent limitation of subunit vaccines (weak CMI). Although the subunit and mRNA vaccines induced similar cellular responses, compared with mRNA vaccines, the subunit vaccine offers practical advantages, including stability and reduced reliance on cold-chain storage, which is critical for broader accessibility, making it a viable alternative for HSV-1 prophylaxis. However, this study has several limitations. The mouse model used in this experiment is not the best model for HSV-1 infection because the infection in the mouse does not become latent or reactivate, which is a hallmark of herpes infection. Therefore, the findings of this study can only reflect the protective effect of the vaccine following primary HSV-1 infection, and it remains unclear whether the cellular immune response elicited by the vaccine completely prevents viral recurrence and whether memory cell immunity is sufficient to eliminate the virus upon reactivation, thereby preventing symptomatic manifestations. Other suitable animal models are needed in future studies to validate the long-term protective effect of the vaccine. For example, a rabbit model of HSV-1 infection could be advantageous. The prominent ocular features of rabbits facilitate an easy visualization of ocular infections and enable efficient tear collection. Furthermore, HSV-1 can establish latency in rabbits, making them an appropriate research model for spontaneous recurrent disease [39]. Future research needs to increase the detection of latency-associated transcripts (LAT) in neural tissues to further validate the vaccine’s protective efficacy. Moreover, it is necessary to optimize the antigen dose to balance the degree of humoral and cellular immunity induced by gC1, gD1, and gE1, and there is also a necessity to evaluate changes in a broader spectrum of cytokines, including IL-4, IL-10, and TNF-α. This would allow a more comprehensive assessment of the type of vaccine-induced immune response, thereby enhancing our understanding of the mechanism underlying immune protection.

## 5. Conclusions

In summary, the results of this study demonstrate that both mRNA and subunit vaccines targeting gC1, gD1, and gE1 can induce protective immunity against acute HSV-1 infection. With its favorable immunogenicity and logistical benefits, the subunit vaccine warrants further investigation as a candidate for preventing HSV-1-associated diseases, including recurrent lesions and neurological complications. Future work should explore long-term protective efficacy, latency control, and scalability to advance these formulations toward clinical application.

## Figures and Tables

**Table 1:** Grouping of vaccines and dosing of the three antigens.

Vaccine Group	gC1 + gD1 + gE1	gC1 + gD1 + gE1 mRNA	CpG 1018S	QS-21	Injection
1. PBS	-	-	-	-	I.M.
2. Subunit vaccine	5 μg each protein	-	5 μg	5 μg	I.M.
3. mRNA vaccine	-	5 μg each pathogen	-	-	I.M.

- Not added; I.M., intramuscular injection.

**Table 2:** gG primer and probe sequences.

Name	Sequence
Forward primer	TCCTSGTTCCTMACKGCCTCCCC
Reverse primer	GCAG/ideoxyI/CAYACGTAACGCACGCT
TaqMan probe	FAM-CGTCTGGACCAACCGCCACACA-BHQ1

**Table 3:** Clinical symptoms during the acute phase after HSV-1 infection in mice.

Groups	Death	Activity Drop	Keratitis
mRNA vaccine	0/6	0/6	0/6
Subunit vaccine	0/6	0/6	0/6
Positive control	2/6	4/4	4/4
Blank	0/6	0/6	0/6

## Data Availability

All the data used during this study are available from the corresponding author upon request.
